# Nanophotonics-inspired all-silicon waveguide platforms for terahertz integrated systems

**DOI:** 10.1515/nanoph-2021-0673

**Published:** 2022-02-08

**Authors:** Ratmalgre A. S. D. Koala, Masayuki Fujita, Tadao Nagatsuma

**Affiliations:** Information Photonics Group, Div. Adv. Electronics & Optical Science, D348 Graduate School of Engineering Science, Osaka University, 1-3 Machikaneyama, Toyonaka, 560-0043 Osaka, Japan

**Keywords:** communications, integration, photonic crystal, silicon photonics, terahertz, waveguide

## Abstract

Recent advances in silicon (Si) microphotonics have enabled novel devices for the terahertz (THz) range based on dielectric waveguides. In the past couple of years, dielectric waveguides have become commonplace for THz systems to mitigate issues in efficiency, size, and cost of integration and packaging using metal-based waveguides. Therefore, THz systems have progressively evolved from cumbersome collections of discreet components to THz-wave integrated circuits. This gradual transition of THz systems from numerous components to compact integrated circuits has been facilitated at each step by incredible advances in all-Si waveguides allowing low-loss, low dispersion, and single-mode waveguiding operation. As such, all-Si waveguides position themselves as highly efficient interconnects to realize THz integrated circuits and further large-scale integration in the THz range. This review article intends to reevaluate the evolution stages of THz integrated circuits and systems based on all-Si waveguides.

## 1 Introduction

A lot of research effort has been dedicated to the terahertz (THz) range in recent years. The THz range is a gold mine in terms of available spectral bandwidth yet to be exploited [1] and covers the frequencies from around 100 GHz to 10 THz. These are higher frequencies associated with higher channel capacities as dictated by the Shannon theorem. Hence, it can be conceivable to achieve data rates as high as one terabits/s in wireless communications [2]. Such ultrahigh-frequency together with broad bandwidths could open the doors for other applications such as space exploration [3, 4], nondestructive and noninvasive imaging [5], and sensing [6], in addition to communications [7].

However, the full potential of the THz spectrum has yet to be realized. A noticeable challenge that hinders the development of technologies and applications in the THz range is the lack of high-power sources and high-sensitivity detectors. This arises from the inherent difficulty of generating THz waves with artificial sources [8]. It is desirable to seek efficient integration with diverse components and sources to compensate for the decrease in available power. This will require THz range interconnects, which have been restricted to metal-based transmission lines for a long time. The metal-based approach has led to bulky THz systems with numerous individual components associated with high insertion and ohmic losses. This has motivated research for all-dielectric waveguides platforms in replacement for metal-based waveguides to reduce the loss. All-silicon (Si) waveguides inspired by the recent progress of Si photonics [9], [10], [11], [12] specifically have been of particular interest. Indeed high-resistivity intrinsic Si has proven to yield low-loss [13], low-dispersion waveguides combined with strategic design considerations. Photonic crystals [14], [15], [16], [17], [18], which are typical photonic nanostructures, are examples of built-in all-Si waveguide platforms, where the wave confinement and waveguiding function are based on the photonic bandgap (PBG) effect [19]. The first notion of THz integrated circuits based on an all-Si platform was proposed in 2012 by Fujita et al. [20]. They reported a photonic crystal slab for the THz waves integrated circuit. In their work, they illustrated an integrated frequency-domain multiplexing transceiver based on photonic crystal slab. Meanwhile, photonic crystal structures have been ideal for realizing high-Q compact cavities employed for sensing applications [21, 22]. These results are considered to be the first generation (1G) of THz integrated circuits. Subsequently, in 2016 Yata et al. presented a fully realized two-channel photonic crystal diplexer for THz wave applications [23] that inspired further integration work towards the realization of a multichannel transceiver built-in photonic crystal. Despite promising results, the realized transceivers had a relatively limited bandwidth, which motivated further investigations to increase the absolute bandwidth, including the array approach as reported in [23].

Other approaches sought to replace photonic crystal waveguides with more efficient structures, including effective medium (EM) waveguides [24] and unclad waveguides [25, 26]. EM waveguides and unclad waveguides are based on total internal reflection (TIR) rather than PBG to achieve in-plane confinement and guiding of THz-wave. This approach yielded a broader bandwidth operation. In addition, metal packaging was developed to support the waveguide platforms [27, 28]. Hybrid integration [29] with active devices such as resonant tunneling diodes (RTDs) yielded communications modules for THz fiber communications [30, 31]. Developed broadband waveguides have enabled broadband components for nondestructive imaging applications [32, 33]. Progress made up to this point can be designated as the second generation (2G) of THz integrated circuits.

By leveraging the technologies achieved in 1G and 2G, it is possible to achieve much larger-scale integration that will efficiently combine sources and detectors such as RTD and photomixers and passive components such as antenna and couplers and guiding components such as hollow waveguides and fibers. Such integration will enable a wide range of applications such as wireless communications, sensing, imaging, and radars in a single compact all-Si platform that can be fabricated from a simple and cost-effective etching process. This will be the third-generation (3G) of THz integrated circuits. Two leading technologies will include topological photonic waveguides, which yield very low loss for waveguide bends and low dispersion [34]. Novel backside coupling technique shows promises for array applications [35].

This review article intends to explore the different stages of THz range integrated circuits and the waveguide technologies that have enabled them. We will begin with THz systems before the integration era by focusing on waveguiding techniques leading to photonic crystal slabs and 1G THz integrated circuits. Here, we will provide a review of THz waveguides, focusing on the operation principle and design of photonic crystal waveguides. The second section will cover more evolved THz waveguides, including EM waveguides and unclad waveguides at the center of 2G THz integrated circuits. In the last section, we discuss large-scale integration toward the realization of 3G THz integrated systems. Here we will discuss the challenges and the potential of the 1-THz band and related technologies.

## 2 The road to THz integrated circuits

In this section, we intend to explore waveguides for the THz range preceding the hybrid integration era. This includes metallic hollow waveguides, planar transmission lines, polymer fiber, photonic crystal waveguides, etc. We look into the performances of each waveguide in terms of key metric including bandwidth, loss and integrability for the realization of THz-range integrated components and systems.

### 2.1 THz waveguides: a review

A key performance index for a waveguide is its propagation loss which is highly correlated to the geometry and the material properties. Waveguides made of metal are associated with significant ohmic losses as the wavelengths are shorter in the THz range [36], [37], [38]. Therefore, dielectric materials are preferred to metal. However, waveguides made of dielectric materials are also associated with losses, mostly due to dielectric absorption of the THz waves. In comparison, high-resistivity Si exhibits low loss and has been the material of choice for highly efficient THz waveguides.

**Figure 1:**
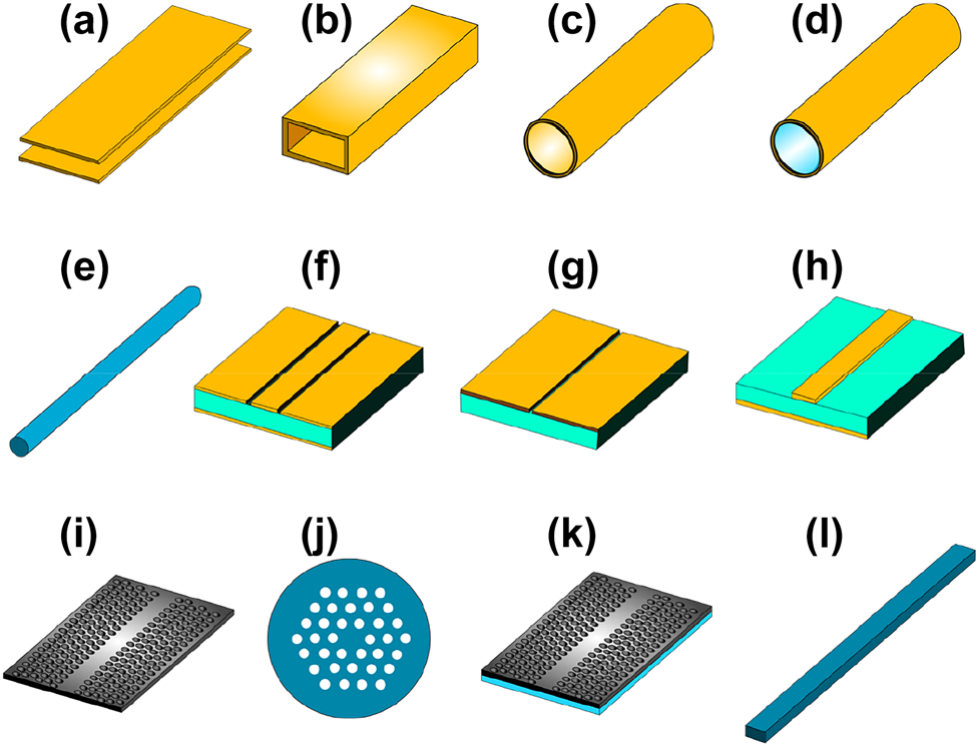
Low-loss waveguides for the THz range, (a) parallel plates waveguide, (b) metallic rectangular waveguide, (c) metallic circular waveguide, (d) circular waveguide with dielectric coating, (e) polymer fiber, (f) coplanar showing substrate in aquamarine color, (g) slot line, (h) stripline, (i) photonic crystal waveguide, (j) photonic crystal fiber, (k) SIIG with dielectric substrate, (l) ribbon waveguide. The colors gray, yellow, and blue represent silicon, metal, low-index dielectric material, respectively.

For a long time, metallic waveguides such as hollow waveguides [39], [40], [41] have been a standard for interconnecting with external power sources, testing, and packaging for the THz range. This is because waveguides in the THz range are built upon well-established waveguides in the microwave region, such as the metallic rectangular and circular waveguide [42]. As most metallic waveguides, metallic hollow waveguides rely on guided wave by metallic media (GMM) principle, i.e., the waves are confined within the metallic walls of the waveguide. Hollow waveguides yield good performance in the microwave region, but when extended to the THz region, the physical size of these waveguides is significantly reduced. This renders foundry processing difficult as extreme accuracy is sometimes required for manufacturing. Another difficulty associated with the physical size of the metallic waveguides in the THz range is the difficulty of interconnecting with planar components of a different scale, generally leading to a modal mismatch at the interface with the hollow waveguide. In most cases, a matching structure that can progressively convert the modes from the large aperture of the metallic waveguide to a much small aperture is needed. Besides, the nonplanar profile of these waveguides makes it difficult to achieve compact packaging. This is a crucial hindrance for THz integrated circuits. More design considerations have to be taken. In addition, because ohmic losses are increased with metal material, the overall performance of the THz system built using such waveguide can be significantly reduced, knowing that there is a lower available power in the THz region compared to the microwave region. The reported loss for WR2.8 waveguide (710 × 355 µm) is ∼0.2 dB/cm for the lower limit of the WR2.8 band 260 GHz and ∼0.4 dB/cm for the upper limit 400 GHz [43]. Parallel-plate waveguides have been widely used for the generation and transmission of THz waves. They consist of two conducting plates positioned closely together. These waveguides offer single-mode operation. Reported propagation loss was 0.3 dB/cm in 0.1–4 THz [44]. Other waveguides include dielectric-coated hollow metallic waveguides. These waveguides also suffer from increased ohmic losses in the THz range. However, propagation loss has been reduced by employing dielectric coating on the metallic waveguide’s inner walls, as reported in [41, 45], [46], [47], [48], [49], [50], [51]. The reported propagation loss of 0.01 dB/cm was achieved in [41]. The dielectric coating allows the electric fields to vanish at the interface with the wall gradually. In contrast, the fields do not vanish at the wall’s interface for metallic rectangular and circular waveguides but penetrate the metal wall where absorption occurs. The loss can be further reduced by optimizing the thickness of dielectric cladding. Increasing the thickness of the cladding translates into reducing the inner diameter of the waveguide, allowing modal matching with THz pulses and higher coupling can be achieved between the dominant mode of the waveguide and propagating THz waves [52]. The increase of the coupling efficiency with the dominant mode is associated with a reduced coupling efficiency with higher-order modes. A further reduction of coupling with higher modes eventually leads to the suppression of those modes and further to a single-mode operation that is ideal for a waveguide. Polymer fibers physically resemble the dielectric hollow waveguide as they present a circular profile. A noticeable difference is the diameter of the waveguide. In the case of polymer fiber waveguides, the diameter is subwavelength, i.e., the diameter is smaller than the operating wavelength. The waveguiding is analogous to that of the circular metal waveguide, and the losses here are a combination of radiation loss and material absorption. The radiation loss originated from nonuniform variations of the diameter, whereas absorption loss results from the polymer material. A propagation loss at 300 GHz was reported as low as 0.01 dB/cm [53]. It is particularly difficult to manufacture fiber with a uniform diameter in the THz range. The diameter for such fiber can be as small as the tens of microns scale for mid to higher THz frequencies. THz waveguides with a more planar profile have been extensively employed. This includes transmission lines such as coplanar, microstrip, and stripline transmission lines. The interest in these waveguides for the THz range has not been greatly significant because these transmission lines are associated with high dispersion and attenuation [36]. The electromagnetic waves leak both in the substrate and the air for microstrip and coplanar transmission lines. Propagation loss for coplanar, microstrip and stripline waveguides can be as high as 10 dB/cm, 15 dB/cm, and 6 dB/cm, respectively [54]. Photonic crystal waveguides are an example of waveguides for the THz range with a planar profile. But contrary to the traditional transmission lines, photonic crystal waveguides have no substrate. Therefore, substrate absorption loss is suppressed. In addition, strong in-plane confinement is achieved because of the PBG effect, yielding very small leakage into the air. Photonic crystal waveguides with extremely low propagation loss have been recently reported [13, 55, 56]. We cover photonic crystal waveguides in-depth as they are essential for THz integrated circuits, as we intend to demonstrate. Noteworthy waveguides built upon photonic crystal structure include photonic crystal fibers and substrate integrated image guides (SIIG). In the case of photonic crystal fibers, they can be viewed as optical fiber with a photonic crystal core. As such, the guiding mechanism is the PBG effect. Early reports of such waveguides go back to 1996 with the report of single-mode silica photonic crystal fibers for light-wave region [57] and have since been efficient ways to generate THz waves [58]. In the case of SIIG, their structure is composed of a dielectric core and two low refractive index porous walls on each side of the core. Consequently, their guiding mechanism is TIR. SIIG with propagation loss as low as 0.35 dB/cm was reported in [59]. Ribbon waveguides also rely on TIR as a guiding mechanism. Their structure is simple and only comprises high-index dielectric core such as graphene core [60]. Such structures have reported a propagation loss of 0.087 dB/cm when operating 100–130 GHz. We provide illustrations for these waveguides in Figure 1, and Table 1 summarizes each waveguide’s performance.

**Table 1:** Performances of various THz waveguides.

Reference	Waveguide type	Frequency (GHz)	Propagation loss (dB/cm)	Confinement principle	Integrability
[44]	Parallel plates waveguide	100–4000	<0.3	GMM	No
[43]	Hollow rectangular waveguide	750–1100	1.92–1.35	GMM	No
[41]	Circular coated hollow waveguide	–	0.01	GMM	No
[53]	Polymer fiber	320–350	<0.01	TIR	Yes
[61]	Microstrip line	Up to 1000	43.4	GMM	Yes
[36]	Coplanar waveguide	Up to 1000	65	GMM	Yes
[54]	Slot line	Up to 1200	26	GMM	Yes
[13]	Photonic crystal	324–361	<0.1	PBG	Yes
[62]	Photonic crystal fiber	171–352	∼18	PBG	Yes
[63]	Ribbon	100–140	0.087	TIR	No
[59]	SIIG	85–105	0.35	TIR	Yes

### 2.2 Photonic crystal slabs and their wave confinement

**Figure 2:**
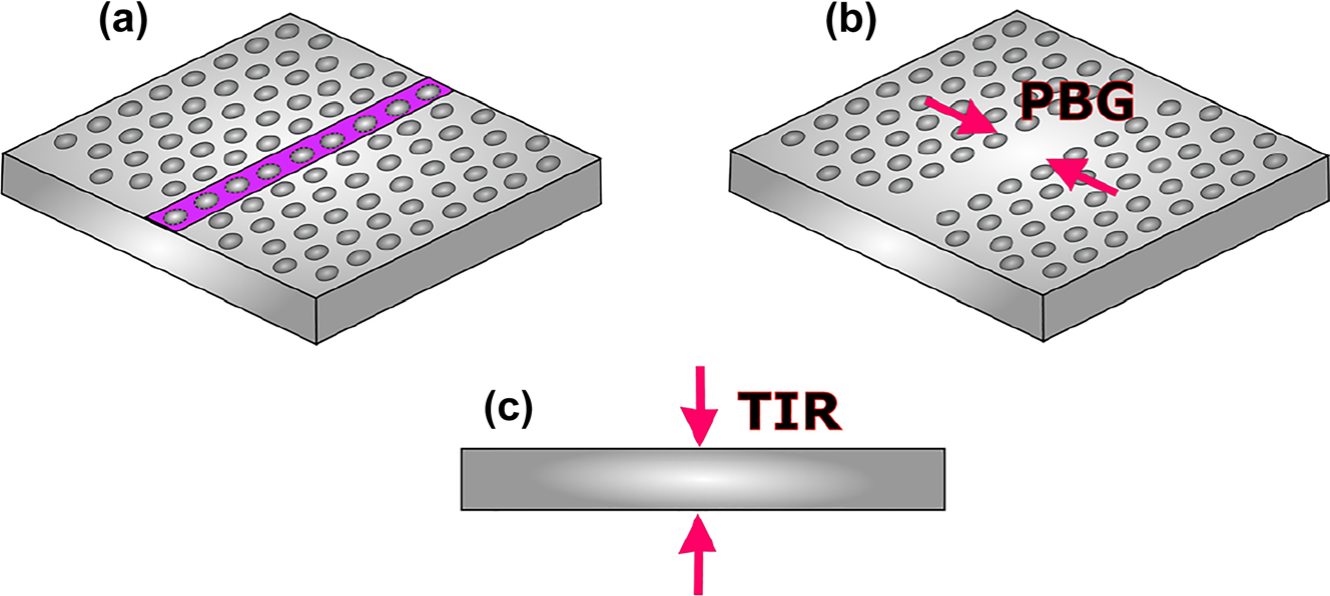
THz waves in photonic crystals, (a) photonic crystal slab showing PGB effect and removable holes (purple region) to create waveguide track, (b) photonic crystal waveguide showing PBG effect and removed holes to create waveguide track, (c) illustration of TIR.

After realizing the potential of the THz range, research efforts have been dedicated to making the most out of what appears to be an endless spectral bandwidth. However, challenges related to the generation, manipulation, and transmission of THz waves arose and forced researchers to seek effective methods for controlling THz waves. The solution to this problem was found in photonic crystal slabs. Photonic crystal slabs are two-dimensional (2D) structures made of semiconductor material through which an array of holes is perforated, as illustrated in Figure 2. Consequently, the resulting structure exhibits a stop-band, also known as PBG, where the electric field is oriented parallel to the slab plane and where no transverse-electric (TE) fields exist because the array of holes created a periodically varying refractive index in the structure. For the components made of photonic crystals, the wider the bandgap, the better it is for applications requiring large bandwidth. The size of the bandgap can be controlled by the arrangement of the holes of the array. Indeed, there is quite a degree of freedom in designing photonic crystal waveguides. With the desired bandgap size as a design starting point, different arrangements of the array of holes can be found to meet that criterion. Key design parameters for photonic crystals include the lattice constant, i.e., the distance between the centers or two adjacent holes and the hole diameter. The methods for finding the right values for *a* and *d* varies from simple parameter sweeps to more advanced algorithm [64, 65]. The first occurrences of photonic crystals go back to 1973 when the periodic structures in integrated optics were reported [66], [67], [68]. Photonic bandgaps, as described, provide a degree of control of THz waves, as they can be engineered to block out THz waves for a given frequency range. The PBG can also be used to further the control and manipulation of THz waves. In 2005, Fujita et al. demonstrated the concept of simultaneous inhibition and redistribution of spontaneous light emission in photonic crystal slab [69, 70]. Indeed, there are two optical modes in a photonic crystal slab: “slab modes” and “vertical modes”. Slab modes are confined in the 2D photonic crystal slab plane by satisfying TIR for the vertical direction. In contrast, vertical modes do not satisfy TIR and are leak out of the slab. Manipulating these modes allows control of spontaneous light emission in 2D photonic crystals. Specifically, with a photonic crystal structure incorporating a single quantum well that emits light with a TE polarization parallel to the slab plane, the spontaneous rate decreases due to the PBG effect. In contrast, the emission rate into the directional normal to the slab plane increases via energy redistribution. Reduced spontaneous emission is associated with an increase of redistribution by the same amount in the PGB region as reported in [69]. This implies that 2D photonic crystals can effectively be used to control THz waves. Subsequently, in 2012, Kakimi et al. demonstrated that THz waves could be trapped into a photonic crystal slab [71]. Trapping and controlling THz waves in photonic crystals can be achieved by leveraging the resonance state. The resonance state is reached when the lattice constant of the photonic crystal is equal to the wavelength in the medium. THz waves normally incident to the plane of photonic crystal slab in the resonance state are trapped inside the photonic crystal, and the in-plane resonant mode is excited. The trapped wave then gradually leaks out of the photonic crystal.

However, the degree of wave confinement in the photonic crystal slab depends on the material absorption of photonic crystal [71]. With the appropriate amount of absorption, THz waves can effectively interact with the absorption and decay in the slab and not leak. This provides another means to control trapped THz waves in photonic crystal slabs. Integrated circuits at this stage can be viewed as the 0th generation of THz integrated circuit components. The choice of photonic crystal slabs to implement interconnect for THz integrated circuit components is also motivated by the possibility of fabrication process in one single micro-electro-mechanical system (MEMS). An entire THz integrated circuit component can be fabricated in an all-Si platform from a single etch process, reducing production costs. However, advanced components such as waveguides must be integrated into photonic crystal slabs to achieve all-Si THz integrated circuit circuits and systems.

### 2.3 Photonic crystal THz Si waveguides

Photonic crystal waveguides are realized by creating a line defect into photonic crystal slabs. Precisely, an entire row of through-holes is filled with semiconductor material to create a path for the waves to propagate, creating a waveguide. The waveguiding principle for photonic crystal waveguides translates into field confinement that is dependent upon PBG. In TE bandgap region, the propagation of THz waves with in-plane polarization is inhibited. Early reports on photonic crystal waveguides go back to 1999 when light transmission in photonic crystal waveguides at light-wave region [19] was first observed. Subsequently, linear waveguides and waveguide bends were introduced in [72], [73], [74], [75] around 2000. Until 2006, the pioneering work on 2D photonic crystal waveguides for fluid sensing by Hasek et al. demonstrated photonic crystal waveguides for practical applications such as sensing. Indeed, there is extensive literature exploring PBG and its potential for manipulating THz waves but not as much on a proof of concept for real-life applications. In their work, Hasek et al. provided experimental proof of concept to theories on measurements methods for fluids and deoxyribonucleic acid (DNA) for sensing application in the healthcare industry [76, 77] using a 2D photonic crystal waveguide implemented in a high-density polyethylene [78]. However, it is noted that other photonic crystal structures, such as the parallel plates photonic crystal waveguides, were also widely used [79].

Such structures are metallic and are bounded by parallel plates in the vertical direction and by a square lattice of holes in the horizontal axis. This is because parallel plates have widely been used to demonstrate efficient guiding of THz waves with little distortion. However, parallel plate waveguides are less efficient at a higher level of integration, and given that they are made of metal, the losses are quite significant. In addition, the beam can only travel in one direction, hence giving very little control. Therefore, spatial confinement and directional control can be achieved by combining a 2D photonic crystal waveguide with parallel plates. In the meantime, as progress on photonic crystal slabs and photonic crystal waveguides was being made, there was still a crucial issue to address, namely the loss in photonic crystal waveguides. In 2009 Li and Zhao reported a Si photonic crystal waveguide with the lowest propagation loss of 9.9 dB/cm at 280 GHz in 270–330 GHz [80]. In 2013, Tsuruda et al. demonstrated a photonic crystal waveguide with reported loss as low as 0.2 dB/cm for both straight waveguides and waveguide bends [13, 56] for 0.315–0.329 THz. Such low loss was achieved by employing a 200 µm-thick high resistivity (20 kΩ) Si combined with proper lattice constant and hole diameter values. The bending degree is an additional design parameter to consider achieving low propagation loss. In general, it is preferable to seek a gradual transition of the waves propagating through the bend. Subsequent research on photonic crystal waveguides reported low propagation loss across an improved bandwidth with high resistivity intrinsic Si. In 2015, Otter et al. introduced variable attenuators and resonators into the photonic crystal waveguide [81]. By illuminating the waveguide with a laser light to generate free carriers in Si waveguide, the propagation loss of a photonic crystal waveguide can be increased so that it behaves as an attenuator. Other defects, such as L3 defects, i.e., removing three adjacent air holes, can create a compact cavity in photonic crystal slabs. Such cavity resonators are essential for applications such as sensing, which has been a key target application for the THz range [82], [83], [84], [85], [86]. Although many resonators were realized for the millimeter waves, they were mostly made of metal, as reported in [87, 88]. In addition, most millimeter-wave resonators did not have a planar form factor. For THz integrated systems, resonators made of low-loss materials and having a planar profile are better suited.

### 2.4 The first generation of THz integrated circuits on all-Si platform

High-resistivity all-Si photonic crystal slabs have proven to be effective for integrating THz range components, including straight waveguides, waveguide bends, grating couplers [89] but also attenuators and resonators. A combination of such components can lead to more advanced devices for novel applications. Indeed, as reported in [20], a multichannel transceiver that implements photonic crystal waveguides and waveguide bends can be realized. It is possible to realize multiple input-output transceivers with photonic crystal waveguides. In which case, each waveguide will correspond to a distinct carrier channel. It is possible to adjust the carrier frequency of each channel by using a resonant cavity tailored to operate at the desired carrier [85, 90]. All channels can afterward be connected using waveguide bends. Such multi-component integrated system applications go beyond multiplexing and are extended to simultaneous sensing/probing for industry-level testing and targeted broadcasting.

**Figure 3:**
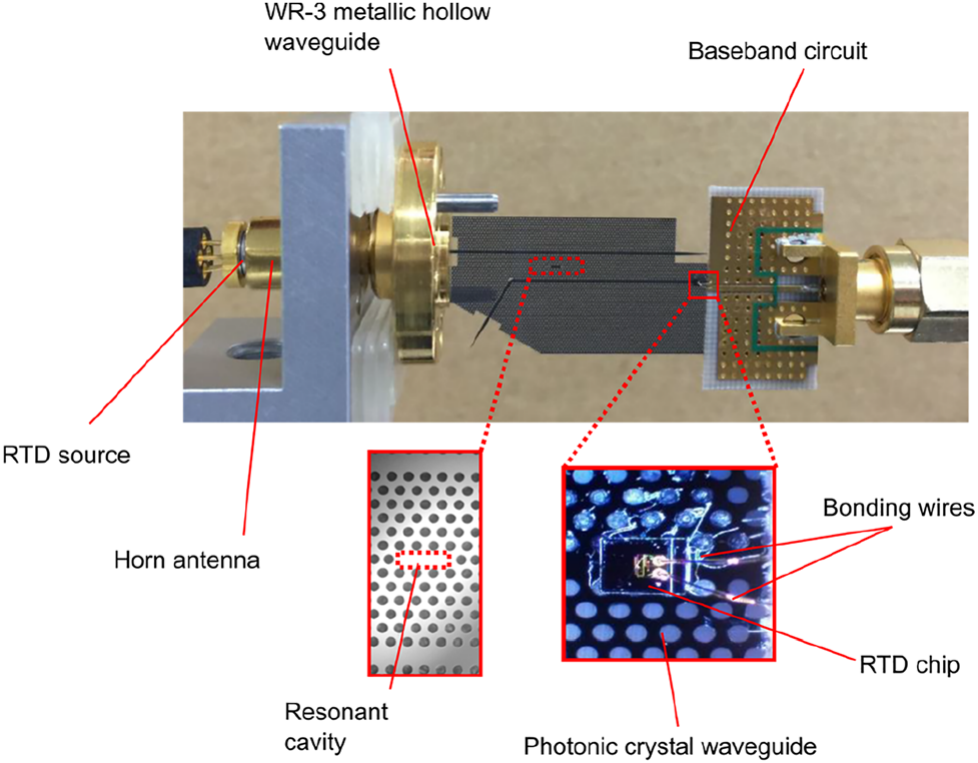
Terahertz sensor realized in all-Si platform. Photonic crystal cavity acts as sensor. This device is representative of the 1G THz integrated systems.

**Figure 4:**
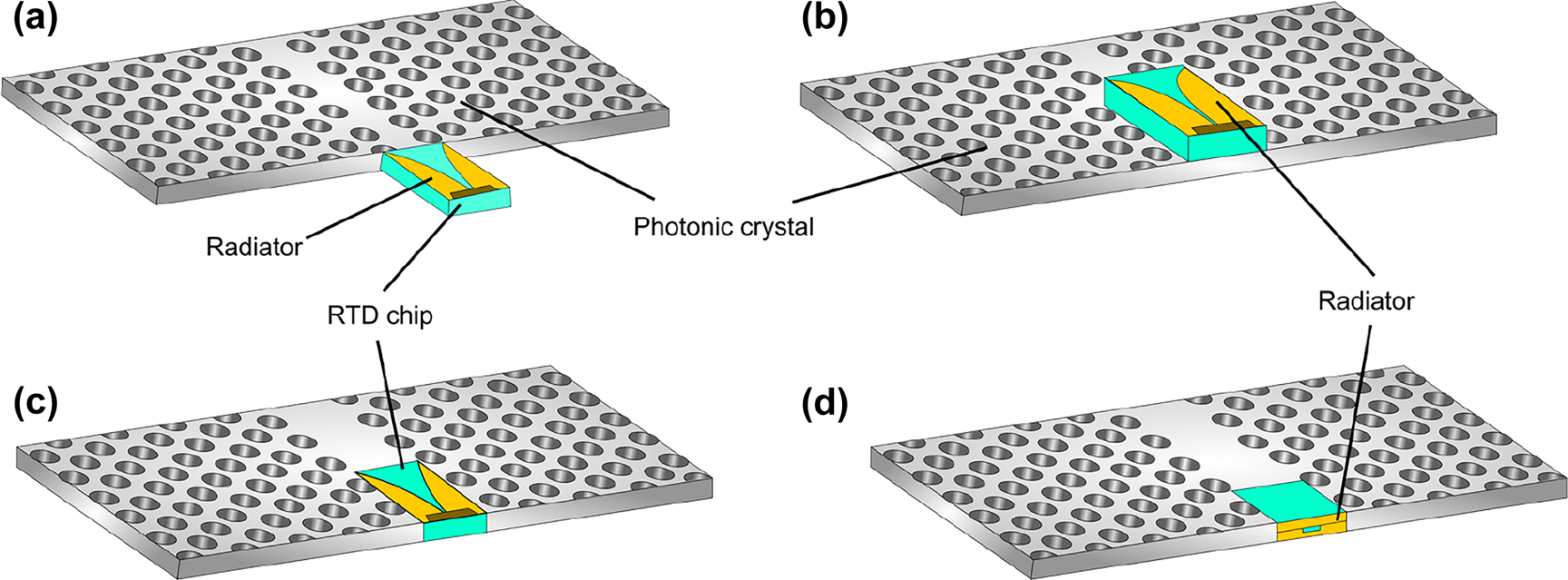
Coupling schemes for hybrid integration with RTD, (a) end-fire coupling, (b) parallel coupling, (c) embed coupling, and (d) backside coupling.

Further integration with oscillators and detectors can enable novel applications. For this purpose, one of the most promising devices is the RTD. RTDs are very compact and can operate as oscillators and detectors at THz bands [91]. The current–voltage graph of RTD exhibits a negative differential conductance (NDC) region. Biasing the RTD with a voltage at the beginning of the NDC makes the RTD device operates as a detector. In contrast, choosing a bias voltage inside the NDC makes the RTD device operates as a fundamental THz oscillator. Photomixer such as uni-traveling photodiodes [92] usually requires two laser sources to generate THz waves and requires physically large components, hence not suited for THz integrated systems at present. In [89], Suminokura et al. reported the integration of RTD with THz photonic crystal waveguides. They reported a tapered slot antenna integrated RTD chip. Different schemes for integrating RTD chips into the photonic crystal waveguide include the end-fire and parallel coupling (Figure 4) [89]. In the case of end-fire coupling, the RTD chip is directly attached to the end of the photonic crystal waveguide to align the tapered antenna with the waveguide track. When the RTD operates as an oscillator, THz waves originating from the RTD are released into the photonic crystal waveguide through the tapered slot antenna. In contrast, for parallel coupling, the RTD rests on a photonic crystal waveguide so that the center of the RTD is aligned with the waveguide track. For an RTD oscillator coupled this way, THz waves are coupled to the waveguide through the chip’s substrate. These coupling schemes directly impact the coupling efficiency, a key performance index for hybrid integrations of this sort. The coupling efficiency indicates how well power is transmitted from the RTD chip to a photonic crystal waveguide and vice versa. End-fire coupling and parallel coupling have reported coupling efficiencies lower than 10%. Which means that there is a lot of power loss and there is a need for better coupling schemes. At this stage, there have been reports of THz integrated systems with passive devices (waveguides, grating couplers) but also active devices (RTDs) built in all-Si platform. In 2017, Okamoto et al. reported a THz sensor based on photonic crystal platform integrated with waveguides, high *Q* (∼10,000) cavity, RTD source and detector. This sensor is representative of 1G THz systems and is illustrated in Figure 3. Key issues to address for 2G THz integrated circuit components include the limited bandwidth of photonic crystal platforms, the low coupling efficiency of active device integration, and practical packaging.

## 3 The second generation of THz integrated systems based on silicon platform beyond photonic crystals

In the previous section, we have established the need for THz integrated systems propelled by all-Si photonic crystal components, including photonic crystal straight waveguides and waveguide bends, diplexers, resonator cavities, and RTDs. However, the integrated systems at that stage yielded a very limited operation bandwidth, and attempts to resolve the bottleneck of the bandwidth issue have not been successful. This section explores more advanced components built upon the pioneering work on photonic crystal slabs and photonic crystal components, including EM waveguides, more recent unclad Si wire waveguides, and their packaging technologies. We will also see how these novel devices have contributed to establishing the second generation of THz integrated systems.

### 3.1 Effective medium waveguides

An EM is a composite material with properties inherited from combining all the properties of the different constituents that make up the composite material. For dielectric effective medium waveguides, there are two constituents, namely the dielectric material and air. This is because EM structures are created by introducing an array of through-holes into a dielectric slab. All-Si EM waveguides have gained popularity because the reduced transmission loss and wideband operation, as reported in [24]. In their work, Gao et al. reported a waveguide core cladded with EM. In comparison with photonic crystal waveguides, the in-plane guiding mechanism in EM waveguides is based on TIR, not on PBG. In addition, the diameter of the holes and the perforation period dimensions are extremely small, which ensures that the resulting structure behaves like a homogenous structure [93]. As a consequence, EM waveguides exhibit an enhanced bandwidth and low dispersion. For the design of the EM structure, a hexagonal lattice can be employed with a lattice constant smaller than the guided wavelength. Because the EM structure is composed of intrinsic Si and air, the resulting effective refractive index is between Si’s refractive index and air’s [93, 94]. As such, larger holes will yield an EM structure with an effective index closer to air, while smaller holes will yield an effective index closer to Si. This creates a 2D index contrast that helps confine the waves within the waveguide core by TIR. The guiding mechanism of EM waveguides is thus solely based on TIR made possible by the high index contrast in both transverse dimensions. However, due to the low cladding index in both planes, i.e., EM cladding and air cladding, EM waveguides typically support two fundamental modes, one parallel to the slab (TE) and another one perpendicular to the slab (transverse magnetic (TM) mode). These modes are associated with electric-fields having distinct relative permittivities that can be approximated using the Maxwell–Garnett approximations and given by the following equations [95]:

$$\varepsilon_{x}=\varepsilon_{\text{Si}}\frac{\left(\varepsilon_{0}+\varepsilon_{\text{Si}}\right)+\left(\varepsilon_{0}-\varepsilon_{\text{Si}}\right)\zeta }{\left(\varepsilon_{0}+\varepsilon_{\text{Si}}\right)-\left(\varepsilon_{0}-\varepsilon_{\text{Si}}\right)\zeta }$$

$$\varepsilon_{y}=\varepsilon_{\text{Si}}+\left(\varepsilon_{0}-\varepsilon_{\text{Si}}\right)\zeta$$

where $\varepsilon_{0}$ and $\varepsilon_{\text{Si}}$ are, respectively, the permittivity of air and Si, and ζ represents the filling factor of the air in Si. The value of the filling factor is dependent upon the pattern of the array of holes. For example, for a square lattice in which the holes are arranged in square patterns, the lattice constant is estimated as $\pi d^{2}/\left(4a^{2}\right)$ , where *d* is the hole diameter, and *a* is the lattice constant, i.e., the distance between the centers of two adjacent holes. And for a hexagonal lattice, the value of the filling factors can be estimated as $\left(\pi d^{2}\right)/\left(2\sqrt{3}a^{2}\right)$ . These values for the filling factor can be deducted from algebraic considerations of the lattices. The choice of the lattice, however, can be motivated by robustness. In which case, the isosceles lattice has been preferred compared to other lattices [55]. In the case of EM cladded dielectric waveguides, additional considerations have to be made. Notably, careful considerations must be taken for the desired propagation mode and the degree of confinement that will impact the waveguide transmission. For the propagating modes supported by the EM waveguides, it is noted that the relative permittivity must be selected to enable single-mode propagation. Notably, lower relative permittivity generates a lower propagation constant which causes the cutoff frequencies of the higher modes to be moved up to higher frequencies in the operation band. Therefore, attention must be paid to choose the right relative permittivity. The realization of broadband THz integrated systems employing EM structures depends on the efficient integration of functional components. Building upon highly broadband EM waveguides, subsequent research reported Bragg filters [96], planar lens [97], [98], [99], [100] and beam splitters [32, 33].

### 3.2 Unclad waveguides

Despite the performance of EM waveguides and their potential for THz integrated circuits, the main hindrance for their use is the difficulty of fabrication associated with the small size of the holes that constitute the EM structure. Indeed, high precision machining would be required to manufacture subwavelength hole diameter. Unclad waveguides were introduced by Headland et al. in 2020 to address this issue [25]. The term “unclad” refers to the absence of cladding as these waveguides are substrate-less and entirely cladded by air. The main motivation is to reduce the complexity of the structure by removing the EM structure. Long before unclad structures, Si on insulator technology was used for THz range waveguides in which the waveguide core was laid on top of an insulator substrate in most cases Si dioxide (SiO_2_) [101, 102]. However, SiO_2_ is typically more absorbent than high-resistivity Si, which renders such waveguides more lossy due to absorption loss of the substrate material.

Intuitively, losses should be reduced by removing a portion, even the entire substrate. Such considerations led to suspended waveguides [103, 104]. Suspended waveguides are micro-scale waveguides entirely substrate less. However, the suspended waveguides are not self-supporting, which is not very practical for physical handling. Photonic crystal structures are usually employed to provide support for suspended waveguides. However, photonic crystal structures have typically limited bandwidth and increased dispersion in the PBG region. This renders suspended waveguides narrowband and dispersive. Subsequent research was oriented toward Si on glass waveguides [60, 103]. However, for THz integrated systems, all-Si components are preferred. Such consideration led to the most recent unclad waveguides. Unclad waveguides have been developed as an extension of EM waveguides by removing the in-plane EM cladding structure and introducing protective Si frame [25]. The resulting waveguide offers more simplicity and greater freedom in choosing crucial design parameters such as waveguide thickness, which translates into faster analysis, simulations, design, and fabrication. Like EM waveguides, unclad waveguides can be fabricated in all intrinsic Si, thus enabling monolithically integration with various functional devices and physical supporting elements entirely made of Si. The appearance of the supporting element can be designed to fit physical packaging and for handling and testing [25].

All-Si unclad waveguides establish a platform for the integration of Y-branches, couplers, and multiplexers. The absence of EM cladding facilitates hybrid integration. Unclad waveguides have reported transmission loss as less than 0.1 dB/cm, making it possible to achieve more complex designs requiring higher and more complex bending. 90-degree-bends Y-branches were realized based on unclad structures as well as evanescent couplers [25]. Structures like resonators were also reported in suspended waveguides using one period of photonic crystal structure [26].

### 3.3 Second generation THz integrated systems

**Figure 5:**
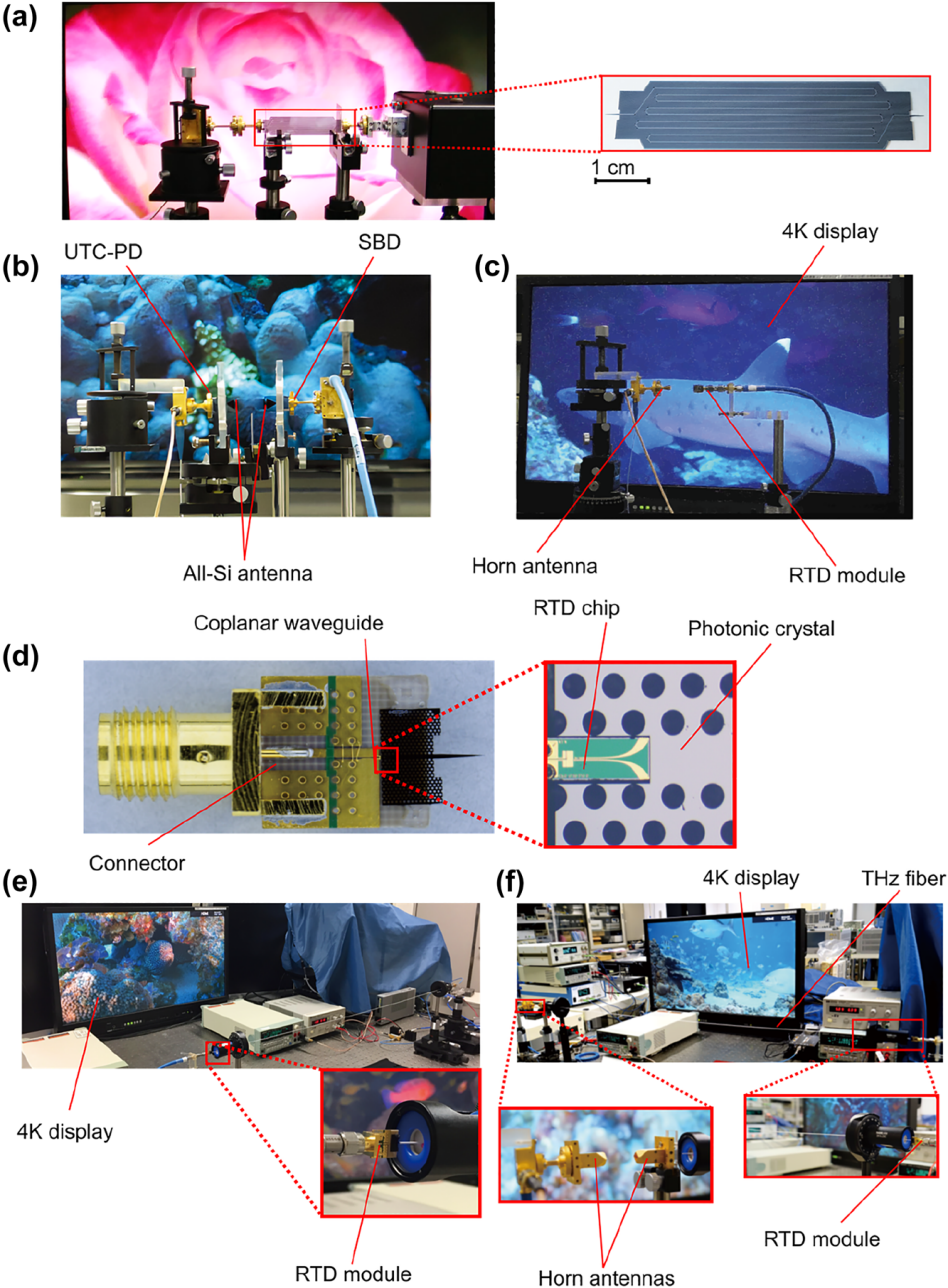
THz communication applications, (a) 4K video transmission over 50 cm-long photonic crystal waveguide [13], (b) 4K video transmission with dielectric rod array antennas [105], (c) wireless transmission of 4K video using a horn antenna and an RTD module shown in (d) [29], (d) RTD integrated module with the RTD chip as inset [30], (e) experimental setup showing the transmission of 4K resolution video over THz fiber wired link [30], (f) 4K resolution transmission over THz fiber wireless link [31].

**Figure 6:**
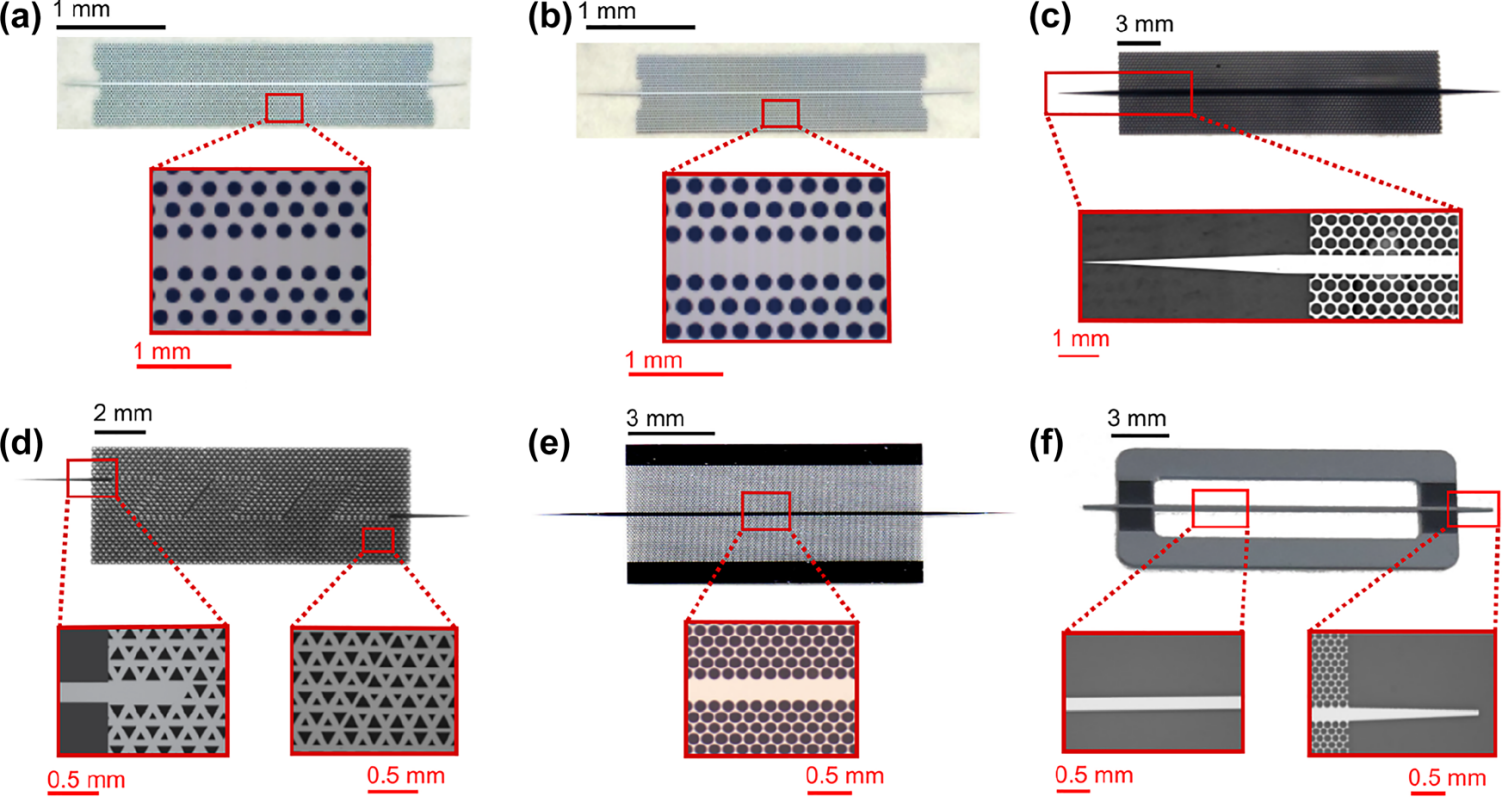
Planar all-Si waveguide platforms and respective simulated field distribution, (a) photonic crystal waveguide with equilateral lattice [108], (b) photonic crystal waveguide with isosceles lattice [108], (c) Bragg-mirror suppressed waveguide [34], (d) topological waveguide [109], (e) EM waveguide [24], (f) unclad waveguide [25].

**Figure 7:**
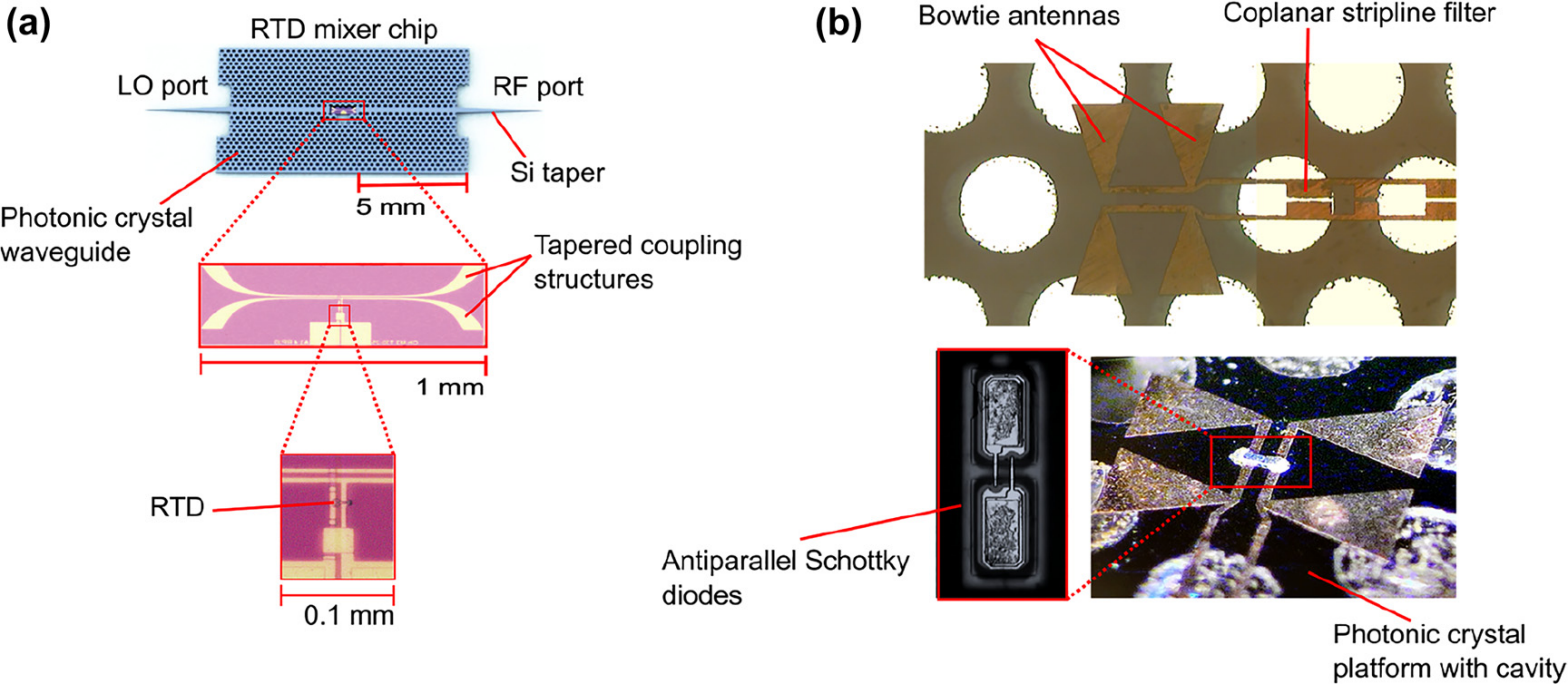
THz mixers integrated into Si photonic crystal platform. (a) RTD [106], (b) Schottky diode [107].

The 1G of THz integrated systems established the potential of such compact systems based on photonic crystal components. The 1G faced some challenges, including the limited bandwidth and the low coupling efficiency for active devices including RTD. Novel devices accelerated the 2G with improved performances in terms of bandwidth. In parallel with the development of EM waveguides and unclad waveguides, a lot of effort sought to improve the integration of RTD and all-Si platforms integration for increased coupling efficiency. Hybrid integration with active components is crucial as a serious hindrance of THz systems is the lack of available power. In 2018, Yu et al. presented a compact Yagi–Uda and tapered-slot coupling structures for the integration of RTDs with the main goal of improving previously reported coupling efficiency (<10%) and 5 GHz bandwidth [20, 21, 85]. Both coupling structures were built on InP substrate. This way, THz waves radiation from the antenna are coupled to the photonic crystal waveguide through the chip’s substrate. This is because the permittivity of the substrate is higher than that of the air. Improved coupling efficiency was achieved with maximum efficiencies of ∼56% and 50% for Yag–Uda and tapered slot antenna, respectively. A 3-dB bandwidth of 41 GHz was realized for the tapered slot antenna against a 3-dB bandwidth of 11 GHz for the Yagi–Uda antenna. The limited bandwidth of the Yagi–Uda structure is attributed to the components, namely the directors, the dipole, and the reflector, which are innately resonant components. On the other hand, the tapered structure with its exponential profile operates as a progressive matching structure between the large size photonic crystal and the small size of RTD. The coupling efficiency of the tapered slot structure can be further increased by improving the exponential profile of the taper, such as to realize a mode converter as was reported in [29]. The improved coupling efficiency of 90% was reported by employing the metallic mode converter. The 3-dB bandwidth was also improved up to 50 GHz. These improvements are also attributed to an improved coupling scheme, notably the embed coupling, as illustrated in Figure 4(c). THz range integrated communication modules were realized employing this coupling scheme, as shown in Figure 5(d). These modules incorporated a dielectric taper as an input/output interface which can be used for coupling with hollow metallic waveguides for testing or wired and wireless THz fiber communications, as illustrated in Figure 5(e) and (f) [30, 31]. Figure 5(a)–(c) show additional THz range communication application employing a straight photonic crystal waveguide [13] (Figure 6), a horn antenna and RTD module [29]; and a dielectric rod array antenna, [105] respectively. Silicon integrated mixers on a Si photonic-crystal platform have been also reported as shown in Figure 7 for 0.3-THz band using RTD Figure 7(a) [106] and 0.2-THz band based on Schottky diode Figure 7(b) [107].

**Figure 8:**
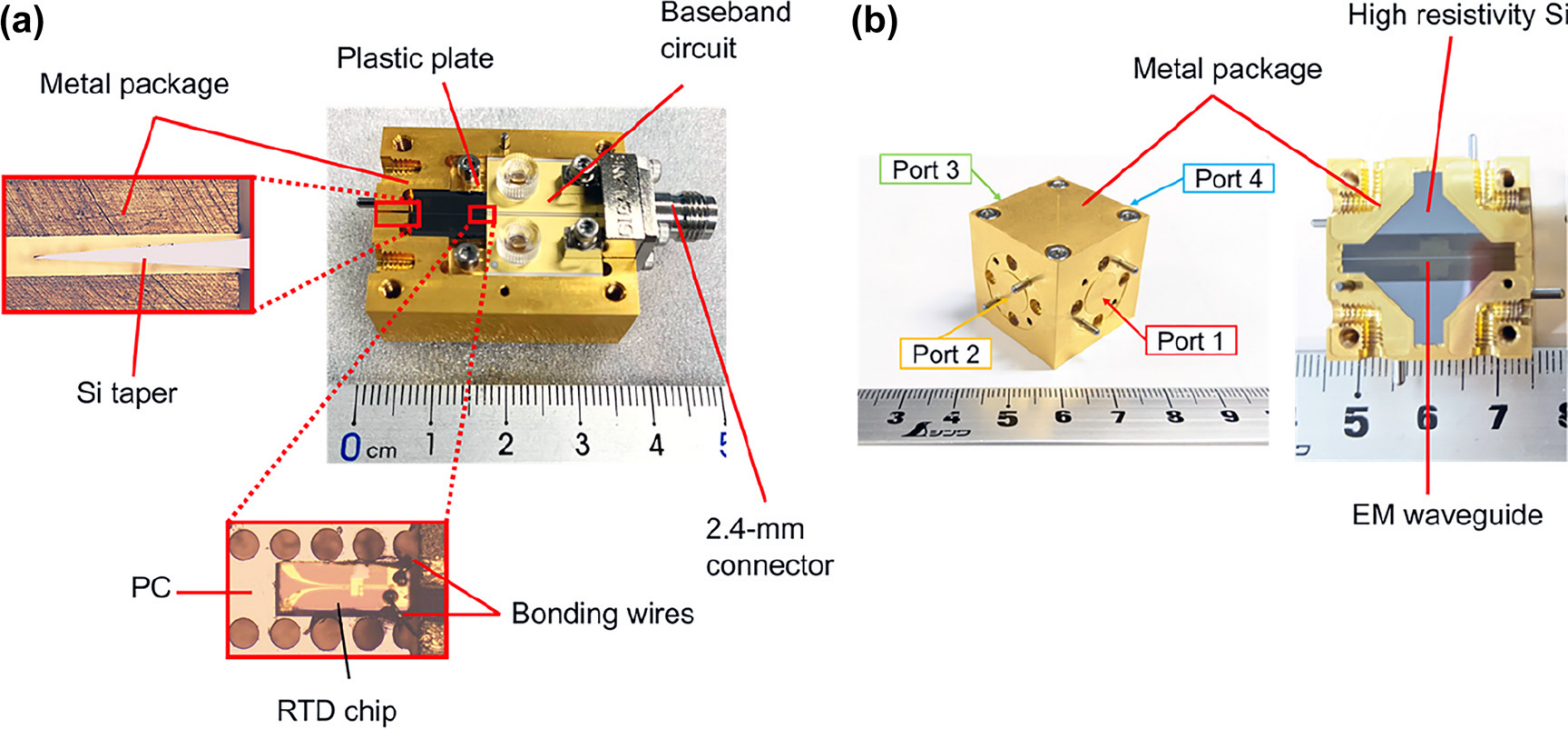
Packaged THz modules, (a) RTD integrated module with photonic crystal waveguide [28], (b) straight waveguide module [27].

THz integrated systems will need compact packaging structures even with improved passive components and highly efficient THz sources. Fortunately, sturdy, compact metal-based packaging structures for THz range waveguide platforms have been reported, as illustrated in Figure 8 [27, 28]. For the design of packaging structures, metal is preferred because of ease of manufacturing for devising requiring high precision machining. Alternative materials such as PEEK (Polyether Ether Ketone) have been gaining increased attention to reduce the metal footprint on the environment. But the stage of development of such material is still at its earliest, and it is challenging to achieve accurate machining. Packaging for waveguide components was reported in [27, 28] for all-Si waveguide platforms, but the employed packaging strategy can easily be extended to diplexers, Y-branches, planar and nonplanar antennas. This marks the 2G of THz range integrated systems. With the possibilities the 2G THz integrated systems offer, various applications related to all fields such as healthcare, transportation, and security could change the way we live in the upcoming years. Exploring such applications is the next frontier of THz integrated systems. A comparison of all Si waveguides and their performances is provided in Table 2, and Figure 6 provides pictures of reported all-Si planar waveguides.

**Table 2:** Comparison of planar all-Si dielectric waveguides.

Reference	Waveguide type	Operation frequency range (GHz)	Relative bandwidth (%)	Reported propagation loss (dB/cm)	Demonstrated applications
[78, 85]	Photonic crystal	97–109	∼11.6	–	Communications (1.5 Gbit/s), HD video transmission, sensing
[83]	Photonic crystal	100	–	–	Liquid sensing
[55]	Photonic crystal	324–361	∼10	<0.1	Communications (36 Gbit/s)
[24]	EM	260–400	>40	0.05	Communications (30 Gbit/s), 4K video transmission
[34, 110]	Topological	320–350	∼8	<0.1	Communications (108 Gbit/s), 4K video transmission
[25]	Unclad	260–390	∼40	0.059	Communications (30 Gbit/s), sensing
[26]	Suspended	500–750	∼40%	0.065	–
[27]	EM	500–750	∼40%	–	Communications (10 Gbit/s)

## 4 Summary and future perspectives

**Figure 9:**
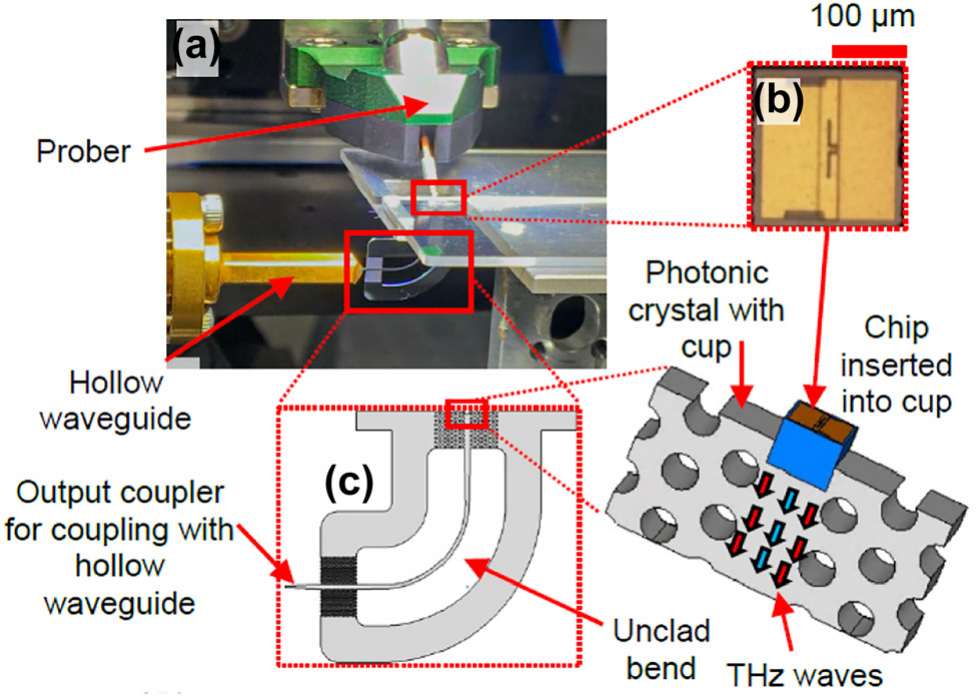
Backside coupled RTD hybrid integration with photonic crystal platform, (a) experimental setup for characterization showing unclad waveguide bend as inset, (b) slot antenna RTD chip.

This review article summarizes the evolution of THz range integrated components and the technologies that have made the integration possible. In particular, we have detailed recent advances made on planar all-Si platforms as “THz silicon photonics”, focusing on waveguides platforms. The starting point was a photonic crystal slab, which allowed trapping and controlling THz waves, leading to photonic crystal waveguides. Photonic crystal waveguides rely on the PGB effect for wave confinement, and recent progress reported photonic crystal waveguides with low propagation loss and low dispersion with limited bandwidth. Photonic crystal waveguides served as a canvas for 1G of THz integrated circuit components. Subsequently, EM waveguides sought to increase the limited bandwidth of photonic crystal waveguides, leading to waveguide with over 40% relative bandwidth. Most recently, unclad waveguides exhibited enhanced bandwidth, low loss, and versatility. With their enhanced performances, these structures were used for THz integrated components, establishing 2G THz integrated circuits. The third generation (3G) for THz integrated components will be oriented towards large-scale integration. The two driving technologies will be the robust topological waveguides [34] and the backside coupling for active devices [35] which is, illustrated in Figures 4(d) and 9. Table 3 provides a summary of generations of THz integrated components.

**Table 3:** Summary of THz integrated technologies based on Si waveguides.

Generation	Waveguide technology	Hybrid integration of active device	Demonstrated application	Comments
First generation	Photonic crystal	Parallel coupling	THz sensor [86]	Early stages of THz integrate components based on photonic crystals
		End-fire coupling		
Second generation	Effective medium	Embedded coupling	THz fiber communications [30, 31]	High-performances waveguides for enhanced THz IC components
	Unclad		THz imaging [32, 33]	
			Wireless communications [35]	
Third generation	Photonic crystal	Embedded coupling	On-going	Large scale integration based on all-Si platform
	Effective-medium	Backside coupling		
	Topological	Novel coupling scheme anticipated		
	Unclad			

**Figure 10:**
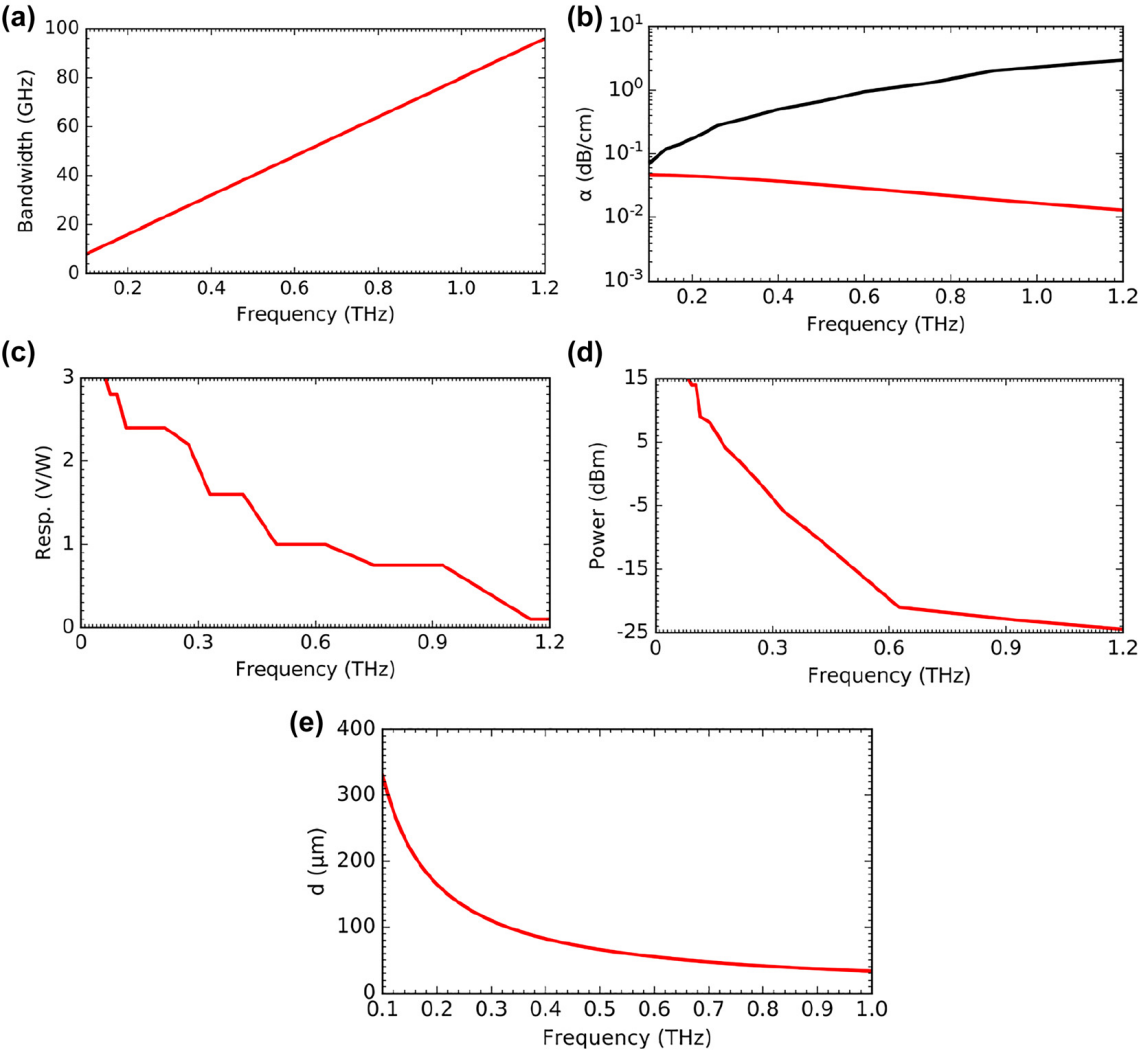
Advantages and challenges for the 1 THz band, (a) absolute bandwidth as a function of the frequency for the unclad waveguide, (b) attenuation as a function of the frequency of high resistivity silicon (red) and metal (black), (c) resp. of ZBD, (d) output power of signal generator and (e) hole diameter of Unclad waveguide as a function of frequency.

Higher data rates would be required for further advanced applications of THz band, implying that higher bandwidths are required for future systems. This could be achieved by increasing the operation frequency for example, to 1 THz-band from 0.3-THz band. As the operation frequency increase, the absolute bandwidth also increases. Figure 10(a) shows the possible absolute bandwidth as a function of frequency for unclad waveguides. Unclad waveguides exhibit increased bandwidth with the frequency. The improvement of the absolute bandwidth will also enable the higher resolution sensing/imaging applications [111] which can be used in healthcare for single-strand DNA detection [112] or in security for enhanced detection of concealed weapons [113]. Another noticeable advantage of the 1 THz band could be the reduction of propagation loss as the operation frequency increases for all-Si components, i.e., as the frequency increases, the absorption loss due to free carrier Si could be decreased [114]. In contrast, the loss associated with metal-based components increases with the operation frequency [43]. This phenomenon is illustrated in Figure 10(b), emphasizing the superiority of high-resistivity intrinsic Si material compared to metal.

Despite the attractiveness of the 1-THz band, great challenges have to be addressed to take full advantage of the untapped spectral bandwidth. Namely, the physical size of the devices in the 1 THz band becomes small. For example, the photonic crystal platform reported in [81] has a lattice constant of 240 µm and a hole diameter of 72 µm for a 0.3-THz band. Using the scaling law, we can estimate the lattice constant and the hole diameter for the 1 THz band at ∼80 µm and ∼24 µm, respectively. These are small dimensions that require higher accuracy machining during the fabrication process. Otherwise, fabrication errors are likely to lead to poor performance of the device. Such stringent requirements are even more exacerbated with unclad waveguides with EM structures. For example, the EM waveguide reported in [24] has a lattice constant *a* of 100 µm and a diameter *d* of 90 µm for the 0.3-THz band. This translates to a lattice constant *a* of ∼33 µm and a hole diameter *d* of 30 μm at 1 THz band, hence a hole separation of 3 µm. Figure 10(e) shows the hole diameter as a function of the frequency for unclad structures. It can be observed that the hole diameter *d* reaches *d* < 50 μm at 1 THz. Establishing high precision machining coupled with new waveguiding techniques will be one of the next research axes for THz integrated components to transition from 2G to 3G. Figure 10(c) and (d) shows the trends of output power and responsivity of existing signal generators based on multipliers, and ZBD (zero biased Schottky diode) detectors as reported by Virginia Diodes Inc. [115, 116]. It can be observed that operation at higher frequencies will be challenging due to the limited output power and low responsivity. It will be crucial to seek alternative higher power sources. These challenges also highlight the future research axes in the field of THz silicon photonics in general.

For large-scale integration, various components will be required. This includes the high power THz sources that could be an array of THz diodes for power combining [117]. Having the higher THz sources can help improve the responsivity of THz detectors as a higher power is injected into as local oscillator signals. This emphasizes the importance of array structures for future THz systems as alternative solutions to limited signal generators and detectors. The research on array configurations for such active devices is still at its earliest stage. Therefore, this is a research axis that can have a great impact on the future. Especially, coherent oscillation by synchronized arrayed THz oscillators for various advanced applications including multilevel modulation communications, high resolution sensing, imaging and ranging. Backside coupling technique is promising for array of active devices. The coupling schemes employed in 1G and 2G of THz integrated systems included parallel, end-fire, and embed coupling. A much more recent coupling scheme, namely the backside coupling, might be at the center of 3G THz integrated systems. The backside coupling scheme is illustrated in Figure 4(d). It can be observed that the major difference with embed coupling is the way the substrate is in direct contact with the Si platform, i.e., the antenna is oriented in the same plane as the Si platform. This is because on-chip antennas innately radiate downwards through their substrate. This has been proven with lens-coupled on-chip antennas [118] that can efficiently extract power from the chip as the lens is put in direct contact with the high-index substrate, allowing index matching to occur at the interface substrate-lens [119]. End-fire antennas have been widely used for the integration of RTDs with all-Si waveguides [29]. However, because end-fire antennas innately radiate forward and THz waves are naturally attracted downwards towards the substrate that has a refractive index higher than that of air, the resulting coupling efficiency employing such antennas was typically limited. This motivated research for alternative coupling schemes to take advantage of the natural way THz waves are attracted downwards in an RTD chip with a semiconductor substrate. Consequently, the chip is inserted backward in the Si waveguide for backside coupling to maximize the power flow between RTD and all-Si waveguide. This coupling technique was validated earlier in 2021, employing the experimental setup illustrated in Figure 9. For the implementation, a broadband unclad waveguide bend was employed. Experiments revealed maximum coupling efficiency of ∼75%. This coupling efficiency can be further improved with appropriate antenna design considerations. This coupling scheme widens the scope of active devices that can be integrated with all-Si waveguide platforms.

A major motivation that led to 2G was the improvement of the limited bandwidth of THz integrated systems built upon photonic crystals. Indeed, the limitation of the bandwidth of such components is attributed to the limitation of the PBG itself. In addition, it is not common for devices built upon PBG to exploit the full PBG for operations such as wireless communications. This is because the behavior of most components is frequency-dependent. As a consequence, the performance of the device is quite nonuniform across the entire operation bandwidth; hence the operation is limited to just a portion of the PBG where performance is the best. As such, it is preferable to seek other types of structures, not relying on PBG to take advantage of the full available bandwidth. Topological photonics has emerged as a promising technology for alternative structures. THz topological photonics find their origins in the topological phase of light [120, 121]. Early reports on topological photonics have demonstrated photonic topological insulators (PTIs). The development of PTIs has been facilitated by the absence of backscattering [122, 123].

Inspired by PTIs, subsequent efforts have developed valley Hall photonic crystals (VPCs) and their practical applications. VPCs are ideal structures for reflection-less waveguides that are of great interest for THz integrated systems. They are likely to yield reduced loss without backscattering and low dispersion as they can be built in high resistivity Si. In 2020, Yang et al. reported the first experimental demonstration of the topological phase of THz wave for on-chip communication [34]. Their work employed VPC and successfully demonstrated near-unity transmission over the operation band, even with structure incorporating sharp bends. Such performance of VPCs is attributed to topological valley kink states. Valley kink states allow robust topological transport of THz waves even across sharp bends. In addition, valley kink states are linear in the bandgap [34], translating into linear dispersion in VPCs. This is indicative of a very little signal delay at different frequencies, hence broadband operation. VPCs also support single-mode operation. It has been observed that only one kink state was found in the bandgap. It is noted that topological bandgap and photonic crystal bandgap are quite different. The former originates from Bragg reflections, and the second originates from the breakage of the inversion symmetry. The difference between photonic crystals and topological photonics like VPC extends beyond the origin of their respective bandgaps. The structure of photonic crystal and VPC is quite different. Typical photonic crystal is created by introducing an array of through-holes arranged mostly in a triangular pattern into a Si slab. In contrast, VPC is created using holes of different sizes arranged in hexagonal [120, 123]. Valley kink states in topological waveguides with linear dispersion and single-mode operation have outperformed typical photonic crystal waveguides in operation bandwidth and overall loss. Record data rates of 108 Gbit/s were reported in 2021 by Webber et al. for 10-mm topological waveguides [110]. This highlights the potential of topological structures for high data rates communication applications. To achieve higher data rate, the increase of the bandwidth of PBG of VPC will be a crucial future subject.

Other research axes include the development of phase shifters and variable attenuators. These components are lacking in the 0.3-THz band or the higher frequency THz bands, and their research needs to be accelerated with a focus on hybrid integration with all-Si planar platforms. Antennas with a beam steering function will be required for advanced wireless applications. Efficient and versatile input-output interfaces beyond hollow waveguide packaging with three-dimensional integrated technology like “LEGO^®^” will open novel unexplored applications. This constitutes another potential research axis toward the realization of 3G THz integrated components.

## References

[1] Song H. J., Nagatsuma T. (2011). Present and future of terahertz communications. *IEEE Trans. Terahertz Sci. Technol.*.

[2] Yang S.-H., Jarrahi M. (2020). Navigating terahertz spectrum via photomixing. *Opt. Photon. News*.

[3] Chen C. P., Hayton D., Samoska L., Dengler R., Mehdi I. (2018). Photonic wireless terahertz wave system for space exploration. *Int. Conf. Infrared Millim. Terahertz Waves*.

[4] Hwu S. U., Desilva K. B., Jih C. T. (2013). Terahertz (THz) wireless systems for space applications. *IEEE Sens. Appl. Symp. Proc.*.

[5] Siegel P. H. (2004). Terahertz technology in biology and medicine. *IEEE Trans. Microw. Theor. Tech.*.

[6] Kakkar A., Schatz R., Pang X. (2015). Impact of local oscillator frequency noise on coherent optical systems with electronic dispersion compensation. *Opt. Express*.

[7] Nagatsuma T. (2011). Terahertz technologies: present and future. *IEICE Electron. Express*.

[8] Williams G. P. (2006). Filling the THz gap - high power sources and applications. *Rep. Prog. Phys.*.

[9] Jalali B., Fathpour S. (2006). Silicon photonics. *J. Lightwave Technol.*.

[10] Soref R., Fellow L., Paper I. (2006). The past, present, and future of silicon photonics. *IEEE J. Sel. Top. Quant. Electron.*.

[11] Bogaerts W., Chrostowski L. (2018). Silicon photonics circuit design: methods, tools and challenges. *Laser Photon. Rev.*.

[12] Siew S. Y., Li B., Gao F. (2021). Review of silicon photonics technology and platform development. *J. Lightwave Technol.*.

[13] Tsuruda K., Fujita M., Nagatsuma T. (2015). Extremely low-loss terahertz waveguide based on silicon photonic-crystal slab. *Opt. Express*.

[14] Noda S., Fujita M., Asano T. (2007). Spontaneous-emission control by photonic crystals and nanocavities. *Nat. Photonics*.

[15] Baba T. (2008). Slow light in photonic crystals. *Nat. Photonics*.

[16] Notomi M. (2010). Manipulating light with strongly modulated photonic crystals. *Rep. Prog. Phys.*.

[17] Noda S., Kitamura K., Okino T., Yasuda D., Tanaka Y. (2017). Photonic-crystal surface-emitting lasers: review and introduction of modulated-photonic crystals. *IEEE J. Sel. Top. Quant. Electron.*.

[18] Asano T., Noda S. (2018). Photonic crystal devices in silicon photonics. *Proc. IEEE*.

[19] Baba T., Fukaya N., Yonekura J. (1999). Observation of light propagation in photonic crystal optical waveguides with bends. *Electron. Lett.*.

[20] Ishigaki T., Fujita M., Nagai M., Ashida M., Nagatsuma T. (2012). Photonic-crystal slab for terahertz-wave integrated circuits. ..

[21] Suminokura A., Tsuruda K., Mukai T., Fujita M., Nagatsuma T. (2014). Integration of resonant unneling diode with erahertz photonic-crystal waveguide and its application to gigabit terahertz-wave communications. ..

[22] Fujita M., Nagatsuma T. (2016). Photonic crystal technology for terahertz system integration. *Terahertz Physics, Devices, Syst. X, Adv. Appl. Ind. Def.*.

[23] Yata M., Fujita M., Nagatsuma T. (2016). Photonic-crystal diplexers for terahertz-wave applications. *Opt. Express*.

[24] Gao W., Yu X., Fujita M., Nagatsuma T., Fumeaux C., Withayachumnankul W. (2019). Effective-medium-cladded dielectric waveguides for terahertz waves. *Opt. Express*.

[25] Headland D., Withayachumnankul W., Yu X., Fujita M., Nagatsuma T. (2020). Unclad microphotonics for terahertz waveguides and systems. *J. Lightwave Technol.*.

[26] Akiki E., Verstuyft M., Kuyken B. (2021). High-Q THz photonic crystal cavity on a low-loss suspended silicon platform. *IEEE Trans. Terahertz Sci. Technol.*.

[27] Shibata N., Uemura Y., Kawamoto Y., Yi L., Fujita M., Nagatsuma T. (2021). 600-GHz-band silicon dielectric waveguide module. *Int. Conf. Infrared Millim. Terahertz Waves*.

[28] Kawamoto Y., Shibata N., Uemura Y. (2021). Integrated resonant tunneling diode with rectangular waveguide I/O using photonic crystal interface. *Int. Conf. Infrared Millim. Terahertz Waves*.

[29] Yu X., Kim J.-Y., Fujita M., Nagatsuma T. (2019). Efficient mode converter to deep-subwavelength region with photonic-crystal waveguide platform for terahertz applications. *Opt. Express*.

[30] Yu X., Miyamoto T., Obata K. (2019). Direct terahertz communications with wireless and fiber links. *Int. Conf. Infrared Millim. Terahertz Waves*.

[31] Yu X., Hosoda Y., Miyamoto T. (2019). Terahertz fibre transmission link using resonant tunnelling diodes integrated with photonic-crystal waveguides. *Electron. Lett.*.

[32] Sagisaka T., Kaname R., Kikuchi M. (2020). Integrated terahertz optics with effective medium for 600-GHz-band imaging. ..

[33] Yi L., Nishida Y., Sagisaka T. (2021). Towards practical terahertz imaging system with compact continuous wave transceiver. *J. Lightwave Technol.*.

[34] Yang Y., Yamagami Y., Yu X. (2020). Terahertz topological photonics for on-chip communication. *Nat. Photonics*.

[35] Koala R., Headland D., Yu X., Nishida Y., Fujita M., Nagatsuma T. (2021). Terahertz RTD chip backside-coupled to photonic-crystal waveguide. *Int. Conf. Infrared Millim. Terahertz Waves*.

[36] Frankel M. Y., Gupta S., Valdmanis J. A., Mourou G. A. (1991). Terahertz attenuation and dispersion characteristics of coplanar transmission lines. *IEEE Trans. Microw. Theor. Tech.*.

[37] Zhang J., Alexandrou S., Hsiang T. Y. (2005). Attenuation characteristics of coplanar waveguides at subterahertz frequencies. *IEEE Trans. Microw. Theor. Tech.*.

[38] Fujishima M., Member S., Amakawa S., Takano K., Katayama K. (2015). Therahertz CMOS design for low-power and high-speed wireless. *IEICE Trans. Electron.*.

[39] Harris D. J. (1979). Waveguides for the 100–1000 GHz frequency range. *Radio Electron. Eng.*.

[40] Gallot G., Jamison S. P., McGowan R. W., Grischkowsky D. (2000). Terahertz waveguides. *J. Opt. Soc. Am. B*.

[41] Mitrofanov O., James R., Fernández F. A., Mavrogordatos T. K., Harrington J. A. (2011). Reducing transmission losses in hollow THz waveguides. *IEEE Trans. Terahertz Sci. Technol.*.

[42] Nordquist C. D., Wanke M. C., Rowen A. M., Arrington C. L., Lee M., Grine A. D. (2008). Design, fabrication, and characterization of metal micromachined rectangular waveguides at 3 THz. ..

[43] Virginia Diodes Inc. ..

[44] Mendis R., Grischkowsky D. (2001). Undistorted guided-wave propagation of subpicosecond terahertz pulses. *Opt. Lett.*.

[45] Matsuura Y., Takeda E. (2008). Hollow optical fibers loaded with an inner dielectric film for terahertz broadband spectroscopy. *J. Opt. Soc. Am. B*.

[46] Bowden B., Harrington J. A., Mitrofanov O. (2008). Low-loss modes in hollow metallic terahertz waveguides with dielectric coatings. *Appl. Phys. Lett.*.

[47] Navarro-Cía M., Vitiello M. S., Bledt C. M., Melzer J. E., Harrington J. A., Mitrofanov O. (2013). Terahertz wave transmission in flexible polystyrene-lined hollow metallic waveguides for the 25-5 THz band. *Opt. Express*.

[48] Doradla P., Joseph C. S., Kumar J., Giles R. H. (2012). Characterization of bending loss in hollow flexible terahertz waveguides. *Opt. Express*.

[49] Navarro-Cía M., Melzer J. E., Harrington J. A., Mitrofanov O. (2015). Silver-coated teflon tubes for waveguiding at 1–2 THz. *J. Infrared, Millim. Terahertz Waves*.

[50] Bowden B., Harrington J. A., Mitrofanov O. (2008). Fabrication of terahertz hollow-glass metallic waveguides with inner dielectric coatings. *J. Appl. Phys.*.

[51] Campion J., Li Y., Zirath H. (2019). Toward Industrial exploitation of THz frequencies: integration of SiGe MMICs in silicon-micromachined waveguide systems. *IEEE Trans. Terahertz Sci. Technol.*.

[52] Zhan H., Mendis R., Mittleman D. M. (2009). Terahertz energy confinement in finite-width parallel-plate waveguides. ..

[53] Chen L.-J., Chen H.-W., Kao T.-F., Lu J.-Y., Sun C.-K. (2006). Low-loss subwavelength plastic fiber for terahertz waveguiding. *Opt. Lett.*.

[54] Grischkowsky D. R. (2000). Optoelectronic characterization of transmission lines and waveguides by terahertz time-domain spectroscopy. *IEEE J. Sel. Top. Quant. Electron.*.

[55] Yu X., Sugeta M., Yamagami Y., Fujita M., Nagatsuma T. (2019). Simultaneous low-loss and low-dispersion in a photonic-crystal waveguide for terahertz communications. *APEX*.

[56] Tsuruda K., Ishigaki T., Suminokura A., Kakimi R., Fujita M., Nagatsuma T. (2013). Ultralow-loss photonic-crystal waveguides for gigabit terahertz-wave communications. ..

[57] Knight J. C., Birks T. A., Russell P. S. J., Atkin D. M. (1996). All-silica single-mode optical fiber with photonic crystal cladding: errata. *Opt. Lett.*.

[58] Argyros A. (2013). Microstructures in polymer fibres for optical fibres, THz waveguides, and fibre-based metamaterials. *ISRN Opt.*.

[59] Patrovsky A., Wu K. (2006). Substrate integrated image guide (SIIG) - a planar dielectric waveguide technology for millimeter-wave applications. *IEEE Trans. Microw. Theor. Tech.*.

[60] Ranjkesh N., Basha M., Taeb A., Safavi-Naeini S. (2015). Silicon-on-glass dielectric waveguide-Part II: for THz applications. *IEEE Trans. Terahertz Sci. Technol.*.

[61] Heiliger H.-M., Nagel M., Roskos H. G. (1997). Low-dispersion thin-film microstrip lines with cyclotene (benzocyclobutene) as dielectric medium. *Appl. Phys. Lett.*.

[62] Wang Y. Y., Wheeler N. V., Couny F., Roberts P. J., Benabid F. (2011). Low loss broadband transmission in hypocycloid-core Kagome hollow-core photonic crystal fiber. *Opt. Lett.*.

[63] Ranjkesh N., Basha M., Taeb A., Safavi-naeini S. (2015). Silicon-on-glass dielectric waveguide — Part II : for THz applications. ..

[64] Shen L., Ye Z., He S., He S. (2003). Design of two-dimensional photonic crystals with large absolute band gaps using a genetic algorithm. *Phys. Rev. B Condens. Matter*.

[65] Meng F., Huang X., Jia B. (2015). Bi-directional evolutionary optimization for photonic band gap structures. *J. Comput. Phys.*.

[66] Elachi C., Yeh C. (1973). Periodic structures in integrated optics. *J. Appl. Phys.*.

[67] Yablonovitch E. (1987). Inhibited spontaneous emission in solid-state physics and electronics. *Phys. Rev. Lett.*.

[68] John S. (1987). Strong localization of photons in certain disordered dielectric superlattices. *Phys. Rev. Lett.*.

[69] Noda S., Chutinan A., Imada M. (2000). Trapping and emission of photons by a single defect in a photonic bandgap structure. *Nature*.

[70] Fujita M., Takahashi S., Tanaka Y., Asano T., Noda S. (2005). Applied physics: simultaneous inhibition and redistribution of spontaneous light emission in photonic crystals. *Science*.

[71] Kakimi R., Fujita M., Nagai M., Ashida M., Nagatsuma T. (2012). Capture of a terahertz wave in a photonic-crystal slab. *Nat. Photonics*.

[72] Chutinan A., Noda S. (2000). Waveguides and waveguide bends in two-dimensional photonic crystal slabs. *Phys. Rev.*.

[73] Johnson S. G., Villeneuve P. R., Fan S., Joannopoulos J. D. (2000). Linear waveguides in photonic-crystal slabs. *Phys. Rev. B Condens. Matter*.

[74] Lončar M., Vučković J., Scherer A. (2001). Methods for controlling positions of guided modes of photonic-crystal waveguides. *J. Opt. Soc. Am. B*.

[75] Lau W. T., Fan S. (2002). Creating large bandwidth line defects by embedding dielectric waveguides into photonic crystal slabs. *Appl. Phys. Lett.*.

[76] Kurt H., Citrin D. S. (2005). Photonic crystals for biochemical sensing in the terahertz region. *Appl. Phys. Lett.*.

[77] Hasek T., Wilk R., Kurt H., Citrin D., Koch M. (2006). Sub-terahertz 2D photonic crystal waveguides for fluid sensing applications. ..

[78] Hasek T., Kurt H., Citrin D. S., Koch M. (2006). Photonic crystals for fluid sensing in the subterahertz range. *Appl. Phys. Lett.*.

[79] Menais R., Grischkowsky D. (2001). THz interconnect with low-loss and low-group velocity dispersion. *IEEE Microw. Wireless Compon. Lett.*.

[80] Li J., Zhao X. (2009). Terahertz waveguides based on photonic crystal. *Opt. InfoBase Conf. Pap.*.

[81] Otter W. J., Hanham S. M., Rider N. M., Holmes A. S., Klein N. (2015). Terahertz photonic crystal technology. *Proc. Workshop on THz*.

[82] Otter W. J., Hanham S. M., Ridler N. M. (2014). 100 GHz ultra-high Q-factor photonic crystal resonators. *Sens. Actuators, A*.

[83] Hanham S. M., Watts C., Otter W. J., Lucyszyn S., Klein N. (2015). Dielectric measurements of nanoliter liquids with a photonic crystal resonator at terahertz frequencies. *Appl. Phys. Lett.*.

[84] Hanham S. M., Ahmad M. M., Lucyszyn S., Klein N. (2017). LED-Switchable high-Q packaged THz microbeam resonators. *IEEE Trans. Terahertz Sci. Technol.*.

[85] Tsuruda K., Okamoto K., Diebold S., Hisatake S., Fujita M., Nagatsuma T. (2016). Terahertz sensing based on photonic crystal cavity and resonant tunneling diode. ..

[86] Okamoto K., Tsuruda K., Diebold S., Hisatake S., Fujita M., Nagatsuma T. (2017). Terahertz sensor using photonic crystal cavity and resonant tunneling diodes. *J. Infrared, Millim. Terahertz Waves*.

[87] Lucyszyn S., Zhou Y. (2010). Characterising room temperature THz metal shielding using the engineering approach. *Prog. Electromagn. Res.*.

[88] Zhou Y., Lucyszyn S. (2010). Modelling of reconfigurable terahertz integrated architecture (Retina) siw structures. *Prog. Electromagn. Res.*.

[89] Suminokura A., Ishigaki T., Fujita M., Nagatsuma T. (2013). Grating coupler for terahertz-wave integrated circuits using a photonic-crystal slab. ..

[90] Yu X., Kim J. Y., Fujita M., Nagatsuma T. (2019). Highly stable terahertz resonant tunneling diode oscillator coupled to photonic-crystal cavity. ..

[91] Asada M., Suzuki S., Kishimoto N. (2008). Resonant tunneling diodes for sub-terahertz and terahertz oscillators. *Jpn. J. Appl. Phys.*.

[92] Nagatsuma T., Ducournau G., Renaud C. C. (2016). Advances in terahertz communications accelerated by photonics. *Nat. Photonics*.

[93] Cheben P., Halir R., Schmid J. H., Atwater H. A., Smith D. R. (2018). Subwavelength integrated photonics. *Nature*.

[94] Mosallaei H., Rahmat-Samii Y. (2000). Photonic band-gap (PBG) versus effective refractive index: a case study of dielectric nanocavities. *IEEE Antennas and Propagation Society International Symposium. Transmitting Waves of Progress to the Next Millennium. 2000 Digest. Held in conjunction with: USNC/URSI National Radio Science Meeting (Cat. No.00CH37118)*.

[95] Subashiev A. V., Luryi S. (2006). Modal control in semiconductor optical waveguides with uniaxially patterned layers. *J. Lightwave Technol.*.

[96] Gao W., Lee W. S. L., Fumeaux C., Withayachumnankul W. (2021). Effective-medium-clad Bragg grating filters. *APL Photonics*.

[97] Headland D., Withayachumnankul W., Yamada R., Fujita M., Nagatsuma T. (2018). Terahertz multi-beam antenna using photonic crystal waveguide and luneburg lens. *APL Photonics*.

[98] Headland D., Fujita M., Nagatsuma T. (2020). Half-Maxwell fisheye lens with photonic crystal waveguide for the integration of terahertz optics. *Opt. Express*.

[99] Headland D., Klein A. K., Fujita M., Nagatsuma T. (2021). Dielectric slot-coupled half-Maxwell fisheye lens as octave-bandwidth beam expander for terahertz-range applications. *APL Photonics*.

[100] Koala R. A. S. D., Headland D., Yamagami Y., Masayuki F., Nagatsuma T. (2020). Broadband terahertz dielectric rod antenna array with integrated half-Maxwell fisheye lens. ..

[101] Zhu H. T., Xue Q., Hui J. N., Pang S. W. (2016). Design, fabrication, and measurement of the low-loss SOI-based dielectric microstrip line and its components. *IEEE Trans. Terahertz Sci. Technol.*.

[102] Amarloo H., Ranjkesh N., Safavi-Naeini S. A. (2018). Terahertz silicon-BCB-quartz dielectric waveguide: an efficient platform for compact THz systems. *IEEE Trans. Terahertz Sci. Technol.*.

[103] Amarloo H., Safavi-Naeini S. (2017). Terahertz line defect waveguide based on silicon-on-glass technology. *IEEE Trans. Terahertz Sci. Technol.*.

[104] Ranjkesh N., Gigoyan S., Amarloo H., Basha M., Safavi-Naeini S. (2018). Broadband single-mode THz suspended silicon-on-glass waveguide. *IEEE Microw. Wireless Compon. Lett.*.

[105] Withayachumnankul W., Yamada R., Fujita M., Nagatsuma T. (2018). All-dielectric rod antenna array for terahertz communications. *APL Photonics*.

[106] Yu X., Ohira T., Kim J. Y., Fujita M., Nagatsuma T. (2020). Waveguide-input resonant tunnelling diode mixer for THz communications. *Electron. Lett.*.

[107] Torres-Garcia A. E., Perez-Escudero J. M., Teniente J., Gonzalo R., Ederra I. (2021). Silicon integrated subharmonic mixer on a photonic-crystal platform. *IEEE Trans. Terahertz Sci. Technol.*.

[108] Headland D., Fujita M., Nagatsuma T. (2019). Bragg-mirror suppression for enhanced bandwidth in terahertz photonic crystal waveguides. *IEEE J. Sel. Top. Quant. Electron.*.

[109] Webber J., Yamagami Y., Ducournau G. (2021). Terahertz band communications with topological valley photonic crystal waveguide. *J. Lightwave Technol.*.

[110] Liang Z., Li J. (2011). Bandwidth and resolution of super-resolution imaging with perforated solids. *AIP Adv.*.

[111] Toccafondo V., García-Rupérez J., Bañuls M. J. (2010). Single-strand DNA detection using a planar photonic-crystal-waveguide-based sensor. *Opt. Lett.*.

[112] Dickinson J. C., Goyette T. M., Gatesman A. J. (2006). Terahertz imaging of subjects with concealed weapons. *Terahertz Mil. Secur. Appl. IV*.

[113] Nagatsuma T., Hisatake S., Fujita M. (2016). Millimeter-wave and terahertz-wave applications enabled by photonics. *IEEE J. Quant. Electron.*.

[114] Virginia Diodes Inc. (2021). ..

[115] Virginia Diodes Inc. (2021). ..

[116] Kasagi K., Suzuki S., Asada M. (2019). Large-scale array of resonant-tunneling-diode terahertz oscillators for high output power at 1 THz. *J. Appl. Phys.*.

[117] Nishida Y., Nishigami N., Diebold S., Kim J., Fujita M., Nagatsuma T. (2019). Terahertz coherent receiver using a single resonant tunnelling diode. *Sci. Rep.*.

[118] Jain R., Hillger P., Ashna E., Grzyb J., Pfeiffer U. R. (2020). A 64-Pixel 0.42-THz source SoC with spatial modulation diversity for computational imaging. *IEEE J. Solid State Circ.*.

[119] Lu L., Joannopoulos J. D., Soljačić M. (2014). Topological photonics. *Nat. Photonics*.

[120] Bahari B., Tellez-Limon R., Kanté B. (2016). Topological terahertz circuits using semiconductors. *Appl. Phys. Lett.*.

[121] Chen W. J., Jiang S.-J., Chen X.-D. (2014). Experimental realization of photonic topological insulator in a uniaxial metacrystal waveguide. *Nat. Commun.*.

[122] Wu L. H., Hu X. (2015). Scheme for achieving a topological photonic crystal by using dielectric material. *Phys. Rev. Lett.*.

[123] Headland D., Yu X., Fujita M., Nagatsuma T. (2019). Near-field vertical coupling between terahertz photonic crystal waveguides. ..

